# Single nucleotide polymorphisms and microsatellites in the canine glutathione S-transferase pi 1 (*GSTP1*) gene promoter

**DOI:** 10.1186/s40575-017-0050-8

**Published:** 2017-10-11

**Authors:** James Sacco, Sarah Mann, Keller Toral

**Affiliations:** Ellis Pharmacogenomics Laboratory, College of Pharmacy and Health Sciences, Drake University, Des Moines, IA 50311 USA

**Keywords:** *GSTP1*, Promoter, Canine, Polymorphism, Microsatellite, Breed, Carcinogen

## Abstract

**Background:**

Genetic polymorphisms within the glutathione S-transferase P1 (*GSTP1*) gene affect the elimination of toxic xenobiotics by the GSTP1 enzyme. In dogs, exposure to environmental chemicals that may be GSTP1 substrates is associated with cancer. The objectives of this study were to investigate the genetic variability in the *GSTP1* promoter in a diverse population of 278 purebred dogs, compare the incidence of any variants found between breeds, and predict their effects on gene expression. To provide information on ancestral alleles, a number of wolves, coyotes, and foxes were also sequenced.

**Results:**

Fifteen single nucleotide polymorphisms (SNPs) and two microsatellites were discovered. Three of these loci were only polymorphic in dogs while three other SNPs were unique to wolves and coyotes. The major allele at c.-46 is T in dogs but is C in the wild canids. The c.-185 delT variant was unique to dogs. The microsatellite located in the 5′ untranslated region (5′UTR) was a highly polymorphic GCC tandem repeat, consisting of simple and compound alleles that varied in size from 10 to 22-repeat units. The most common alleles consisted of 11, 16, and 17-repeats. The 11-repeat allele was found in 10% of dogs but not in the other canids. Unequal recombination and replication slippage between similar and distinct alleles may be the mechanism for the multiple microsatellites observed. Twenty-eight haplotypes were constructed in the dog, and an additional 8 were observed in wolves and coyotes. While the most common haplotype acrossbreeds was the wild-type *1A(17), other prevalent haplotypes included *3A(11) in Greyhounds, *6A(16) in Labrador Retrievers, *9A(16) in Golden Retrievers, and *8A(19) in Standard Poodles. Boxers and Siberian Huskies exhibited minimal haplotypic diversity. Compared to the simple 16*1 allele, the compound 16*2 allele (found in 12% of dogs) may interfere with transcription factor binding and/or the stability of the GSTP1 transcript.

**Conclusions:**

Dogs and other canids exhibit extensive variation in the *GSTP1* promoter. Genetic polymorphisms within distinct haplotypes prevalent in certain breeds can affect *GSTP1* expression and carcinogen detoxification, and thus may be useful as genetic markers for cancer in dogs.

**Electronic supplementary material:**

The online version of this article (10.1186/s40575-017-0050-8) contains supplementary material, which is available to authorized users.

## Background

Glutathione S-transferases (GSTs) are a widely-expressed family of enzymes that play an important role in detoxification by catalyzing the conjugation of many hydrophobic and electrophilic compounds with the reduced form of the tripeptide glutathione (GSH). Substrates for these enzymes include an extensive array of xenobiotics, including drugs, toxins and environmental carcinogens [1]. The conjugation of xenobiotics with GSH is catalyzed by GSTs in the cytosol, endoplasmic reticulum and mitochondria. Based on their biochemical, immunologic, and structural properties, the cytosolic or soluble GSTs are categorized into 4 main classes: alpha (A), mu (M), pi (P), and theta (T). Each class can be subcategorized into various related isozymes.

GST pi 1 (GST-P1) is expressed in extrahepatic tissues such as placenta, lung, gut and erythrocytes [2, 3], where it is involved in the biotransformation of carcinogens such as acrolein [4], and benzo[a]pyrene diol epoxide (BPDE) [5], endocrine-disrupting pesticides such as atrazine [6], and numerous drugs, including cisplatin [7] and chlorambucil [8]. While humans and rats express one subunit belonging to the P1 class of GSTs, GSTP1, mice express two pi subunits, GSTP1 and GSTP2. The situation may be analogous in dogs, who appear to have one annotated *GSTP1* gene, located on chromosome 18 (chr18:49,905,161–49,908,182 on the CanFam3 dog genome assembly), and another uncharacterized “*GSTP*-like’ gene (chr18:49,950,873–49,953,818) adjacent to it.

Polymorphisms in the *GSTP1* gene can lead to impaired GST function. These DNA variants influence susceptibility to carcinogens and the toxic effects of some pesticides, affect the response to chemotherapy used in the treatment of metastatic colon cancer and multiple myeloma, and modify the risk of several forms of cancer. For example, a single nucleotide polymorphism (SNP) in the coding region of human *GSTP1* (c.313 A > G, denoted as rs1695), present in 35% of the population, leads to decreased enzyme activity [9, 10]. This polymorphism results in a Ile105Val change in the GSTP1 enzyme that is, for example, less able to conjugate carcinogens such as BPDE [11]. In fact, this allelic variant has been associated with increased susceptibility to acute myeloid leukemia, prostate, lung, bladder, gastric, oral and breast cancer [12–18]. Genetic and epigenetic variability in the promoter region of *GSTP1* also affects gene expression and influences susceptibility to several types of cancer [19–22].

In dogs (*Canis lupus familiaris*), several studies suggest a possible causal relationship between exposure to certain environmental chemicals and canine cancers. An increased risk of canine malignant lymphoma has been associated with owner application or commercial treatment of lawns with the commonly used herbicide 2,4-dichlorophenoxyacetic acid [23, 24], as well as heterogeneous pesticide mixtures [25]. Living in industrial areas, or in proximity to polluted sites, incinerators, and/or radioactive waste, are also significant risk factors [26, 27]. The expression of GSTs in dogs can vary widely. GST-T activities have been characterized in livers from research beagles, and 12% of these dogs had virtually no GST-T protein expression or activities [28]. In addition, *GSTT1* polymorphisms have been associated with lymphoma in dogs [29]. It is not known if the expression of *GSTP1* shows a similarly wide variability between individual dogs, or whether GSTP1-null allelic variants exist in the population. A preliminary study we have conducted (unpublished data) indicated that, in contrast to the coding region, the canine *GSTP1* promoter sequence contained multiple polymorphisms. By affecting gene expression, these polymorphisms could potentially impact detoxification, and consequently modulate the risk of cancer and other diseases in dogs.

Therefore, the objective of this study was to investigate the variation in the *GSTP1* gene promoter in a diverse population of domestic dogs, compare the incidence of these variants between dog breeds, and predict the potential functional effects of these genetic variants. In order to determine which polymorphisms may have been introduced by domestication or breed formation, we also studied the same genetic region in other species representing the *Canidae* family, the gray wolf (*Canis lupus*), coyote (*Canis latrans*), red fox (*Vulpes vulpes*) and gray fox (*Urocyon cinereoargenteus*).

## Methods

### Animals and sample collection

The 278 unrelated pure-bred dogs (144 males, 134 females; 102 breeds) recruited for this study were either privately owned or provided through the collaboration of Paws & Effect, the Des Moines Kennel Club, and the Des Moines Obedience and Training Club (Additional file 1). Genomic DNA from 37 of these dogs was kindly donated by Lauren Trepanier, University of Wisconsin-Madison, and Michael Court, Washington State University. No dogs were euthanized for the purpose of this study. The American grey wolves (*n* = 14), coyote (*n* = 7) and foxes (*n* = 8) were either privately owned or from the Colorado Wolf and Wildlife Center (Divide, CO), Wolf Park (Battle Ground, IN), Blanke Park Zoo (Des Moines, IA), and the JAB Canid Education and Conservation Center (Santa Ysabel, CA). Buccal cell samples were collected from the dogs using cheek swabs designed for use in canines (Performagene®, DNA Genotek Inc.). DNA collected in this manner is stable for at least one year when stored at room temperature. All experimental procedures were approved by the Drake University Institutional Animal Care and Use Committee.

### DNA sequencing

Following inactivation of nucleases and precipitations of impurities in the buccal samples, genomic DNA was purified via ethanol precipitation. We used 200 ng of this DNA in a 25 μl of reaction mixture containing 0.25 μM of each primer (F: CCACCTCCCTCCTTCCAGTA; R: GCCTTCCAGGAACTCTGACC), 1 μl of GC Enhancer, and 12.5 μL of Amplitaq Gold 360 Master Mix (Applied Biosystems, Foster City, CA) in order to amplify the genomic region from c.-619 to c.1 + 123). After an initial incubation at 95 °C for 10 min, PCR amplification was performed for 40 cycles consisting of 95 °C for 30 s, 57 °C for 30 min and 72 °C for 45 s, followed by a final extension at 72 °C for 7 min. The specificity of each PCR was checked by electrophoresis on a 1.5% agarose gel. Following purification by Exo-SapIT (Affymetrix, Santa Clara, CA), the amplicons were submitted to bidirectional Sanger sequencing using the Big-Dye Terminator v3.1 (Eurofins Genomics, Louisville, KY). Five percent of the samples were randomly chosen and resequenced to confirm the initial genotype result. Sequence assembly and identification of genetic polymorphisms was performed using Staden package software (http://staden.sourceforge.net/).

### Data analysis

Haploview [30] was used to calculate population genetic descriptors as well as to infer linkage disequilibrium (LD) parameters (D’ and logarithm of odds scores, LOD). The chi-squared test for independence was used to assess Hardy–Weinberg equilibrium. Interallelic LD between multiallelic loci and SNPs was estimated by MIDAS software [31]. This program was also used to infer evolutionary relationships between the polymorphisms found.

Multiple comparative DNA sequence alignments between canine and other mammalian *GSTP1* promoter and transcript sequences was performed using Clustal Omega [32] in order to determine the degree to which the sequences are conserved. Pairwise sequence alignment was computed by PromoterWise [33], which allows for inversions and translocations that are common in promoters. The aligning of transcription factors to the canine *GSTP1* promoter sequence was performed by using TRANSFAC position-specific scoring matrix models through the LASAGNA algorithm v 2.0, an integrated webtool for transcription factor binding site search and visualization [34]. By inputting sequence variants, we identified the effects that promoter polymorphisms may have on proteins that may bind to our sequence of interest. RNA secondary structure prediction was carried out using the Mfold program v 3.5 [35]. This program determines optimal and suboptimal secondary structures of RNA calculated for 1 M NaCl at 37 °C, and computes free energy contributions for various potential secondary structures. Estimation of repeat variability (VARscores) within microsatellite sequences was determined by SERV. Larger VARscores correlate with higher predicted repeat variability [36].

## Results

**Fig. 1:**
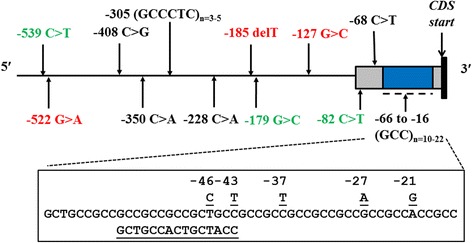
Gene map displaying the position of *GSTP1* promoter polymorphisms in the domestic dog and other canids. Exon 1 is shown as a shaded box, with its 3′end representing the start of the coding sequence, which consists of a single adenine base. Variants found only in dogs are shown in red, while those exclusive to wolves and/or coyotes are in green. Inset shows the DNA sequence for the major microsatellite 17-repeat unit allele and the genetic polymorphisms found within this repeat region; the sequence of the compound repeat associated with certain alleles is shown below this sequence

Fourteen polymorphic loci, including twelve SNPs and two microsatellites were found in the proximal *GSTP1* promoter region (chr18: 49,908,620–49,908,119) of domestic dogs (Fig. 1). The five variant alleles between positions c.-21 and c.-46 were located within the microsatellite sequence on the 5′ untranslated region (5′UTR) in exon 1.

All variants were novel, with the exception of the commonly occurring −46 T > C, −228 C > A, and −350 C > A. These three single nucleotide variations were found in 35–50% of dogs. The previously unreported −21G, −27A, and −43 T alleles were present in 14–36% of dogs (Table 1).

**Table 1:** *GSTP1* promoter polymorphisms and minor allele frequencies (MAF) in domestic dogs, wolves and coyotes

Polymorphism accession no.^a^	Position on dog chromosome 18	Sequence variant	MAF		
			Dog	Wolf	Coyote
ss2019497428	49,908,119	c.-21 A > G	0.356	0.143	0.000
ss2019497429	49,908,125	c. -27 G > A	0.257	0.257	0.429
ss3021042895	49,908,135	c.-37 C > T	0.002	0.000	0.286
ss3021042898	49,908,141	c.-43 C > T	0.138	0.036	0.429
rs22633673	49,908,144	c.-46 T > C	0.496	0.857	0.643
ss2019497431	49,908,166	c.-68 C > T	0.131	0.036	0.214
	49,908,180	c.-82 C > T	0.000	0.143	0.143
ss2019497432	49,908,226	c.-127 G > C	0.009	0.000	0.000
	49,908,277	c.-179 G > C	0.000	0.143	0.071
ss2019497433	49,908,283	c.-185 delT	0.079	0.000	0.000
rs22646524	49,908,326	c.-228 C > A	0.464	0.107	0.143
ss2019497435–6	49,908,403	c.-305(GCCCTC)_2_	0.000	0.143	0.214
		c.-305(GCCCTC)_3_	0.049	0.036	0.000
		c.-305(GCCCTC)_5_	0.002	0.000	0.000
rs22646522	49,908,448	c.-350 C > A	0.379	0.107	0.286
ss2019497438	49,908,506	c.-408 C > G	0.013	0.000	0.286
ss2019497439	49,908,620	c.-522 G > A	0.005	0.000	0.000
	49,908,637	c.-539 C > T	0.000	0.036	0.286

^a^With respect to dog genome

Several polymorphisms appear to be unique or much more frequent in dogs, when compared to their closest relatives, wolves and coyotes. For example, the c.-185 delT variant was found in 8% of dogs but not in the other *Canis* species (Table 1). MIDAS analysis also indicated that this base deletion appears to be the most recently evolved polymorphism in the dog *GSTP1* promoter region. The c.-228 C > A polymorphism, present in almost half the dogs genotyped, was found in only 11% of wolves. At c.-46, the least frequent variant in dogs (C) is the major allele in wolves and coyotes. Similarly, the c.-305 GCCCTC repeat genotype differed between the three groups; in wolves and coyotes, the minor allele is the (GCCCTC)_2_ repeat as opposed to the (GCCCTC)_3_ variant in dogs. A total of 4 variants were only observed in wolves and coyotes.

The 5′UTR microsatellite, located 16 residues upstream of the start codon, was highly polymorphic (Table 2). This tandem repeat consists of a GCT followed by a series of (GCC) triplet units of variable length, with purities ranging from 80.3% (22*1 or 22*2 alleles) to 97.9% (16*1 allele). In dogs, the most common repeats were 16 and 17, occurring in 86% of the study population. The prevalent microsatellite length in the other canids was 18 in wolves, and 10 and 12 in coyotes. The 11-repeat unit motif, seen in 10% of dogs, was not found in their wild relatives. Moreover, several repeat unit lengths appear to be unique to wolves (12, 13, 20), and coyotes (12, 20). Both simple and compound repeats were observed for alleles with 16 and 22 repeat units. For example, a (GCC)_5_ stretch (from −54 to −37) in the 16 repeat allele is replaced by GCTGCCACTGCTACC. The compound 16-repeat allele containing this sequence, referred to as 16*2, was observed in a third of dogs (or 12.4% of the entire study population) that had the 16 simple triplet repeat microsatellite allele (denoted as 16*1). The longest 22-repeat allele, which was observed in the heterozygous state in only two dogs, also contains the sequences GCTGCAACTGCTACC(GCC)_2 or 3_ACTGCTACC(GCC)_1 or 2_ (denoted as 22*1 and 22*2 respectively). The 12*2- and 20-repeat alleles, observed in wolves and coyotes, contained a GCTGCCACTGCTACC motif that is identical to the one observed in the 16*2 allele in dogs. This motif was very common in coyotes, being observed in 5 out of the 7 coyotes (3 homozygotes; 2 heterozygotes) that were genotyped.

**Table 2:** 5′ UTR microsatellite repeat length diversity, sequence and occurrence observed in dog (D), wolf (W), and coyote (C)

Repeat unit	Sequence	VAR score	dG (kcal/mol)	D	W	C
10	GCT(GCC)_7_ACCGCC	0.58	−5.6	0.007	0.000	0.286
11	GCT(GCC)_8_ACCGCC	0.75	−8.5	0.099	0.000	0.000
12*1	GCT(GCC)_9_ACCGCC	0.88	−9.5	0.000	0.040	0.000
12*2	GCT(GCC)_3_GCTGCCACTGCTACCGCCACCGCC	0.72	−4.8	0.000	0.000	0.286
13	GCT(GCC)_10_ACCGCC	0.97	−11.5	0.000	0.180	0.000
14	GCT(GCC)_13_	1.09	−14.5	0.013	0.000	0.000
15	GCT(GCC)_14_	1.12	−17.5	0.005	0.000	0.000
16*1	GCT(GCC)_15_	1.13	−17.5	0.302	0.140	0.000
16*2	GCT(GCC)_3_GCTGCCACTGCTACC(GCC)_3_ACCGCCACCGCC	0.58	−7.3	0.124	0.000	0.143
17	GCT(GCC)_5_GCT(GCC)_8_ACCGCC	1.10	−14.4	0.424	0.214	0.143
18	GCT(GCC)_5_GCT(GCC)_9_ACCGCC	1.10	−18.0	0.009	0.285	0.000
19	GCT(GCC)_5_GCT(GCC)_6_ACCGCCACCGCCACCGCC	1.07	−14.4	0.013	0.000	0.000
20	GCT(GCC)_3_GCTGCCACTGCTACC(GCC)_3_ACC(GCC)_3_ACCGCCACCGCC	–	−11.4	0.000	0.140	0.143
22*1	GCT(GCC)_3_GCTGCAACTGCTACC(GCC)_3_ACTGCTACC(GCC)_3_ACCGCCACCGCC	0.64	−11.0	0.002	0.000	0.000
22*2	GCT(GCC)_3_GCTGCAACTGCTACC(GCC)_2_ACTGCTACC(GCC)_4_ACCGCCACCGCC	–	−13.3	0.002	0.000	0.000

Sequences shown are 5′ → 3′ on the (−) strand. The SERV VARScore refers to the consensus GCC sequence. Computed Gibbs’ energy contributions (dG) shown are for the most stable RNA secondary structure motif only

The 5′UTR tandem repeat was predicted to form a hairpin RNA structure at the 5′ end of the GSTP1 transcript. The stability of this secondary structure, as indicated by the Gibbs’ energy (dG), decreases with smaller numbers of tandem repeats. Comparison of alleles of equal length but differing purity (for example 12*1 vs 12*2, and 16*1 vs 16*2) showed that the compound repeat alleles were expected to be half as stable as their simple repeat counterparts, perhaps because the 5′ hairpin loop is missing or is structurally altered (Additional file 2a). This decreased stability appears to extend to the entire *GSTP1* transcript (Additional file 2b). The VARScores indicate that the highest mutation rates, after consideration of the number of GCC repeat units, unit length and repeat purity should occur in the commonly occurring 16*1 and 17 alleles in dogs and the 18 allele in wolves (Table 2). Conversely, the repeats least likely to experience GCC deletions or insertions are the smallest-length 10 repeat and the compound 16*2 and 22*1 repeats.

The gray fox *GSTP1* promoter sequence was more similar to dog (98.2%), than to red fox (96.1%), which in turn showed more similarity to gray fox (96.1%) than dog (95.9%). Alignment of the promoter sequence of red and gray fox with dog showed differences at 16 and 12 loci respectively, with four of these loci shared between the two fox species (Additional file 3). With one exception (the 5′ UTR microsatellite), none of these were located at the sites of the genetic polymorphisms observed in the *Canis* species. In the grey fox, there were two repeating GCCCTC units at the locus which corresponds to c.-305 in the dog; this repeat number was only observed in three wolves and two coyotes but in none of the domestic dogs. In the red fox, two of the four repeating units were changed to GCCCCCGCCCCC. In the 7 red foxes sequenced, no polymorphisms were observed within the entire promoter region, including the tandem repeat. The red foxes were all homozygous for a 12-repeat exon 1 microsatellite (GCC(GCC)_3_ACC(GCC)_3_(ACCGCC)_2_) that was distinct from the equal-length alleles observed in wolves (12*1) and coyotes (12*3) (Table 2). The single gray fox sequenced had a unique 8-repeat/16-repeat microsatellite genotype (GCT(GCC)_7_ACCGCC and GCT(GCC)_3_GCTGCCACTGCTGGCT(GCC)_5_ACCGCC), with a single base (underlined) interrupting the triplet repeat sequence.

Alignment of the ‘wild-type’ GSTP1 transcript (which includes the 17-repeat allele) with other mammalian transcripts showed evidence of the microsatellite in a number of other species, with the highest number of repeats (13) observed in the white-tailed deer and Bactrian camel. The other species all showed repeat numbers lower than 10, with the two rodent species not showing any GCC repeat units in their 5′UTR (Additional file 4). No information is available on whether these sequences are polymorphic, as has been observed in the three canid species described in this study.

Using the genotypes from homozygous dogs, 28 distinct *GSTP1* promoter haplotypes could be constructed in the dog (Additional file 5). Fifteen of these haplotypes were observed only in the heterozygous state and thus could only be inferred. An additional 8 haplotypes were only found in wolves and/or coyotes. Designation of haplotypes was based on the nucleotide sequence of the reference boxer genome assembly (Canfam 3.0), with the wild-type or most common sequence designated as *1A. Haplotype names were first numbered according to the genetic polymorphism pattern in the promoter region upstream of the microsatellite region (that is, upstream from c.-68). Each haplotype was then designated with letters after the number, each letter corresponding to a specific SNP pattern within the microsatellite. Each haplotype name ends with a number in parentheses, which refers to the repeat unit length found associated with each specific allele (Table 3). The common *1A, *2A, and *6A haplotypes were associated with two or more different tandem repeat units (Additional file 5). A similar phenomenon was observed for the *1H haplotype in wolves, with four different repeat lengths (18 and 13-unit predominating) observed occurring with this allelic arrangement. While dogs share five haplotypes with wolves and one with coyotes, there are significant differences. The commonest haplotypes in dogs are *1A(17) and *6A(16) (accounting for almost half of all dogs), while the prevalent alleles in wolf and coyote are, respectively, *1H (17) and *1C(10), which were either absent or occurring at low frequency in the dogs. The *3A(11) haplotype, characterized by the T deletion at −185 and a microsatellite length of 11 repeats, was observed in around 10% of dogs but was not observed in coyotes and wolves. Haplotypes with microsatellite unit lengths greater than 17, while common in wolves and coyotes (haplotype frequencies of 0.393 and 0.142 respectively), were uncommon in dogs (haplotype frequency: 0.029).

**Table 3:** Comparison of *GSTP1* promoter haplotype in dogs, wolves and coyotes

Haplotype	Dog	Wolf	Coyote
*1A (17)	0.257	0.179	0.071
*1C (10)	0.007	0.000	0.286
*1H (17)	0.000	0.286	0.000
*1H (13)	0.000	0.143	0.000
*2A (16)	0.077	0.000	0.000
*3A (11)	0.081	0.071	0.000
*6A (16)	0.218	0.036	0.000
*10A (16)	0.040	0.000	0.000
*11A (16)	0.09	0.071	0.143
*17A(20)	0.000	0.036	0.071
*19A (20)	0.000	0.000	0.071
*20A (17)	0.000	0.000	0.071
*21A (12)	0.000	0.000	0.214
*22A (12)	0.000	0.000	0.071
**Ĥ**	**0.859**	**0.878**	**0.902**

Ĥ: Haplotype diversity (in bold); only haplotype frequencies > 0.05 shown. Number in parentheses denotes 5′UTR microsatellite repeat length

Promoter haplotypes were compared for breeds in which at least five dogs or more were represented (Table 4). A wide inter-breed variation in the type and extent of genetic diversity was observed. Chihuahuas and Beagles had the highest haplotypic diversity (a measure of the uniqueness of a particular haplotype in a given population). On the other hand, 20 out of 22 Boxers, and all the Siberian Huskies, had a *1A(17)/*1A(17) diplotype, resulting in a very low or no haplotypic diversity. The commonest haplotype in dogs, *1A(17), was not found in Basenjis and Dobermans. The -408C > G transversion occurring in 6 out of 11 Standard Poodles (found only in the *8A(19) haplotype), was, with the exception of one Shar-Pei absent in all other dogs, but, interestingly was also found in 3 out of 7 coyotes (*21A and *21B haplotypes). The *3A(11) haplotype was over-represented in older breeds such as Afghan Hounds, Basenjis, and Greyhounds. Haplotypes with the 16*2 compound repeat (*9A, *10A) occurred more often in more modern breeds such as Beagles, Dobermans, and Golden Retrievers.

**Table 4:** Comparison of *GSTP1* promoter haplotype frequencies between different dog breeds

Haplotype	All dogs	Afg	Bas	Sib	Chi	Gre	Box	Lab	Pem	Bea	Dob	Gol	Ger	Poo
*1A (17)	0.257	0.200	0.000	1.000	0.300	0.071	0.932	0.125	0.100	0.115	0.000	0.300	0.545	0.045
*1A (18)	0.004	0.000	0.200	0.000	0.000	0.000	0.000	0.000	0.000	0.000	0.000	0.000	0.000	0.000
*1B (17)	0.086	0.000	0.000	0.000	0.000	0.286	0.023	0.125	0.000	0.231	0.000	0.000	0.091	0.045
*2A (16)	0.077	0.000	0.000	0.000	0.200	0.071	0.000	0.063	0.100	0.038	0.333	0.200	0.000	0.318
*2B(17)	0.013	0.000	0.000	0.000	0.100	0.000	0.000	0.000	0.000	0.000	0.000	0.000	0.045	0.000
*3A(11)	0.081	0.600	0.500	0.000	0.000	0.571	0.023	0.125	0.100	0.000	0.000	0.000	0.000	0.000
*5A(17)	0.011	0.000	0.000	0.000	0.100	0.000	0.000	0.000	0.000	0.077	0.000	0.000	0.000	0.000
*6A(16)	0.218	0.100	0.100	0.000	0.100	0.000	0.000	0.438	0.300	0.269	0.417	0.050	0.182	0.227
*6A(17)	0.027	0.000	0.000	0.000	0.000	0.000	0.000	0.000	0.000	0.000	0.000	0.000	0.136	0.000
*7A(16)	0.005	0.000	0.000	0.000	0.000	0.000	0.000	0.000	0.200	0.000	0.000	0.000	0.000	0.000
*8A(19)	0.013	0.000	0.000	0.000	0.000	0.000	0.000	0.000	0.000	0.000	0.000	0.000	0.000	0.318
*9A(16)	0.072	0.100	0.000	0.000	0.100	0.000	0.000	0.063	0.200	0.154	0.083	0.300	0.000	0.000
*10A(16)	0.040	0.000	0.000	0.000	0.000	0.000	0.000	0.063	0.000	0.077	0.167	0.000	0.000	0.000
**Ĥ**	**0.860**	**0.644**	**0.756**	**0.000**	**0.911**	**0.626**	**0.133**	**0.803**	**0.889**	**0.853**	**0.741**	**0.809**	**0.672**	**0.777**

Only those haplotypes with frequencies higher than 0.01 are shown. *Afg* Afghan Hound, *Bas* Basenji, *Sib* Siberian Husky, *Chi* Chihuahua, *Gre* Greyhound, *Box* Boxer, *Lab* Labrador Retriever, *Pem* Pembroke Welsh Corgi, *Bea* Beagle, *Dob* Doberman Pinscher, *Gol* Golden Retriever, *Ger* German Shepherd, *Poo* Standard Poodle. Ĥ: Haplotype diversity shown in bold

Some uncommon haplotypes, such as *1C (10), were detected in one or two breeds only (3 alleles in two Chow Chows; 1 allele in a Tibetan terrier). Interestingly, this haplotype was also observed in 6 coyotes. The otherwise rare *6A(17) haplotype was seen 5 times in the three New Guinea Singing Dogs investigated, with the other Singing Dog having the commoner *6A(16) haplotype.

The mechanisms by which these polymorphisms could affect *GSTP1* expression via effects on transcription factor binding were investigated using the web-based program LASAGNA-Search. The wolf and coyote −539 C > A SNP may delete the binding sites for ARE86 (TRANSFAC Accession number M00415), Pax-2 (M00486), and C/EBP (M00159) and introduce novel sites for interactions with CHOP:C/EBP alpha (M00249) and STAT4 (M000498), while the 408G variant deletes an ARP-1 (M00155) site and creates a new site for Brachyury protein (M00150) binding. The common 350 C > A and −228 C > A SNPs result in new sites for the binding of NRF-2 (M00108) and GATA (M00076, M00462, M00075) respectively. The rare -127C allele is predicted to result in a new GC box (M00255) and E2 binding site (M00107, M00181). The wolf and coyote −82 C > T, located just inside the 5′UTR, may generate a new PPARalpha:RXR-alpha (M00242) binding site, while the other 5′UTR SNP, −68C > T, also unique to these wild canids, creates new sites for the binding of HNF-4alpha1 (M00411) and COUP-TF (M00158). The Wilms Tumor zinc finger (WT1-KTS) protein (T01839) was predicted to bind to the multiple EGR1 GGCG core sequences (on the + strand) within the microsatellite. Binding sites for this transcription factor were decreased for alleles with a smaller number of repeat units, and in repeats with decreased purity. For example, while there were 25 potential binding sites on the 16*1-repeat allele, there were only 11 sites on the 11-repeat allele, and just 5 sites on the similar size but compound 16*2-repeat allele (Additional file 6). On the other hand, alleles with the GCTGCCACTGCTACC motif (12*2, 16*2, 20, 22-repeats) were predicted to have a new binding site for KLF12 (M00468). In the case of the *22-repeat alleles, the unique GCA triplet within the repeat introduces a binding site for MyoD (M00184) that binds to the consensus TGCAACTGCT.

## Discussion

The promoter region of a gene is essential for its proper function, containing enhancers and repressors that control its rate of expression. Certain promoter features, such a TATA box, *cis* mutations, and the presence of unstable tandem repeats, increase expression divergence, or the capacity of a gene to evolve in expression [37]. Genes containing these type of promoters preferentially diverge when the effects of natural selection are minimized [38], a good example of which is canine domestication. The DNA resequencing performed in this study demonstrated that the canine *GSTP1* promoter region (with a TAATAA box located at c.-102) contains a number of genetic polymorphisms of varying frequency and a highly polymorphic tandem repeat sequence located in the 5′ untranslated region. It is possible that the promoter architecture found in the canine *GSTP1* gene has arisen both to allow flexibility in the biotransformation of existing and new xenobiotics as well as a direct consequence of inbreeding practices.

Molecular phylogeny studies have shown that the dog family, or *Canidae*, can be classified into four clades, including the wolf-like canids (dog, gray wolf, coyote), the fox-like canids (red fox), the South American canids, and the smallest, most primitive clade represented by the grey fox and the island fox [39]. The dog *GSTP1* promoter sequence showed the best similarity with the other *Canis* species, gray wolf and coyote, followed by the gray fox, and red fox. Although sample sizes for the wild canids used in our study were too small for definite conclusions, we can tentatively conclude that our data supports the aforementioned phylogeny studies.

A total of 16 polymorphic loci were identified in the canid *GSTP1* promoter, of which 13 were polymorphic in the dog population, 11 were polymorphic in the wolf, and 12 were polymorphic in the coyote. Almost half the dogs sequenced had the wild-type *1A(17) and the *6A(16) haplotype. Three loci (c.-82, c.-179, c.-539) were seen to have the allele fixed in dogs but polymorphic in both wolves and coyotes, which likely reflects the bottlenecks associated with domestication or breed formation. The c.-46C > T allele was observed in 50% of dogs but only in 14% of wolves and 36% of coyotes, suggesting that this polymorphism may have arisen from domestication, and that the ancestral allele for this SNP (rs22633673) should therefore be ‘C’. Nine different microsatellite repeat lengths were observed in dogs, while six and five repeat lengths were seen in wolves and coyotes respectively. The *3A(11) dog haplotype is characterized by the 11-repeat microsatellite, which is in strong linkage disequilibrium with the deletion variant at c.-185. This haplotype was not observed in wolves and coyotes and appears to have arisen as a consequence of dog domestication. The *3A(11) haplotype may thus prove useful in DNA marker studies seeking to distinguish between domestic dogs and their wild relatives. Another potential marker is the 1H(n) haplotype, which is found at high frequency in wolves but is absent in both dogs and coyotes. Haplotypes which are unique to and found in almost half of the coyotes studied were the *1C (10) and the *21A (12) haplotypes. Interestingly, the *1C (10) was also found in 2 Chow Chows and 1 Tibetan Terrier, both of which are ancient breeds [40] and would conceivably retain more ancestral alleles than the more recent breed groups.

The fox species were not polymorphic at any of the loci common to the *Canis* species, and exhibited sequence dissimilarities at 21 different positions. The lack of variation between the 7 foxes sequenced, including those polymorphisms within the microsatellite region, was surprising in view of the fact that 4 red foxes originated from different geographical areas in the US and the other 3 were Russian domesticated foxes. It would be interesting to further investigate microsatellite variation in a greater number of coyotes, wolves and foxes, as well as other *Canis* and *Canidae* species.

Since most dog breeds represent closed breeding populations that receive limited genetic variation [41], we expected to find significant differences between dog breeds, as well as a lack of variation within these breeds. The *3A (11) haplotype predominated in Afghans, Basenjis and Greyhounds, while the commonest haplotypes in Standard Poodles were *2A(16) and *8A(19), the latter haplotype occurring only in this breed. A lack of genetic diversity in the *GSTP1* promoter was observed in Boxers and Siberian Huskies. Boxers are known to have low levels of heterozygosity, this being one of the reasons why a female dog from this breed was selected to have its genome sequenced at the Broad Institute [42]. Despite the fact that the 6 Siberian Huskies in the study were not related and came from different states within the US, they had identical *1A(17) haplotypes. This lack of genetic diversity is surprising in view of the fact that Siberian Huskies have ancient origins and display high heterozygosity in microsatellite data compared with other breeds [40].

The canine *GSTP1* promoter containsa microsatellite mainly consisting of GCC triplet units tandemly repeated anywhere from 10 to 23 times. This repeat exhibits a very diverse distribution across species, even across closely related species, indicating rapid evolutionary change. Significant expansion of this microsatellite may have occurred in species belonging to the genus *Canis*, as it appears that the corresponding region in other mammals is much shorter. The red fox sequences were not polymorphic, perhaps indicating that the longer repeats occurred with the appearance of extant canids around 10 million years ago. Similarly, a GCC dodecamer repeat found in the coding region of the canine *EPM2* gene has been shown to be polymorphic across the *Canidae* but not to other close relatives of the dog [43].

**Fig. 2:**
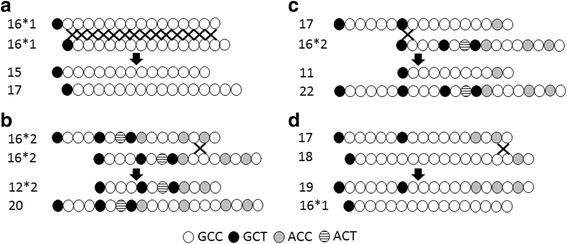
Derivation of *GSTP1* 5′UTR microsatellite alleles by unequal recombination between identical (**a**, **b**) and distinct (**c**, **d**) repeat sequences. Possible point of an exchange is indicated by an ‘X’

The microsatellite in the canine *GSTP1* promoter has a very high GC content, which means that there is a greater tendency to form hairpin-loop and slipped-strand DNA structures during replication, recombination, and repair. These structures result in genetic instability and increased polymorphisms [44]. Two mechanisms are probably the reason for the microsatellite polymorphisms observed. One is replication slippage, which occurs when the daughter strand slips back one or more repeating unit in pairing with the template strand, leading to a trinucleotide expansion or contraction during DNA replication. This probably accounts for alleles which only differ by one repeat unit, as in the 14, 15 and 16*1 repeats seen in the *2A and *6A haplotypes, and possibly even those alleles that differ by two GCC units (for example, 14 and 16*1). The other mechanism is unequal recombination or crossing-over, which is a type of gene duplication or deletion event that deletes a sequence in one strand and replaces it with a duplication from its sister chromatid in mitosis or from its homologous chromosome during meiosis. Based on the microsatellite alleles observed, several types of crossover events can be postulated to occur via intra-repeat and/or inter-repeat recombination mechanisms. The simplest and most common event may involve the exchange of one GCC repeat at multiple sites, generating, for example, the 15 and 17 alleles from two parent 16*1 alleles (Fig. 2a). Identical-length alleles may also recombine only at specific sites on the tandem repeat, as may occur when two 16*2 alleles exchange 4 repeats to form the compound 12*2 and 20 alleles, as was observed in coyotes (Fig. 2b). Unequal exchanges of repeats can occur between alleles with different lengths, as between the 17 and 16*1 alleles to form daughter alleles with 11 and 22 repeats (Fig. 2c), or alleles with 15 and 18 repeats. Whilst the longer allele was observed in only two dogs, the truncated 11 allele was the third most frequent allele observed, probably because the 16- and 17-repeat unit alleles are the most common in the dog. The 19-repeat unit allele seen in standard poodles could be another example of unequal crossing-over between 2 different alleles, in this case the 17 and 18 repeat alleles, which also generates the common 16*1 allele (Fig. 2d). Support for this mechanism comes from the fact that all the standard poodles that were heterozygous for the 19 allele had the 16*1 allele. Moreover, the 16*1 and 19 alleles represent the major forms of the microsatellite in the standard poodles sequenced, with allele frequencies of 0.59 and 0.32 respectively. Finally, an ancestral recombination event could account for the origin of the common dog 16*2 and 17 alleles from crossing-over the 13- and 20-repeat variants that appear to be found only in wolves.

In humans, it has been estimated that a third of promoter variants may alter gene expression to a functionally significant extent [45]. Several studies have demonstrated that tandem GGC or CCG repeats in gene promoters have significant effects on gene function. For example, expansion of a CGG repeat in the 5′UTR of the fragile site mental retardation 1 (*FMR1*) gene causes epigenetic changes that reduce transcription factor binding and are the cause of Fragile X Syndrome [46, 47]. Two-repeat differences in GCC repeat length in a microsatellite located in the thiopurine S-methyltransferase (*TPMT*) 5′UTR may be responsible for ultra-high enzyme activity and consequent resistance to treatment by thiopurine drugs [48]. Tandem repeats can modulate *GSTP1* expression by changing the copy number of binding sites for transcription factors, as has been observed with the binding of Sp1 to the human *EGF* gene [49], the interactions of Sp1 and AP2 to the human *RFC* gene [50], and the binding of p53 to *PIG3* [51]. The *GSTP1* microsatellite GCC repeat polymorphisms were predicted to affect the number of potential binding sites for the Wilms Tumor zinc finger (WT1-KTS) protein. The Wilms tumor gene 1 (WT1) is a transcription factor that acts as an oncogene in acute myeloid leukemia [52], and a recent Chip-Seq investigation has revealed that binding of the WT1-KTS isoform in leukemic cells is correlated to active transcription of numerous genes [53]. The 16*2 allele, which was found in 12% of the dogs in the study and over-represented in breeds such as Pembroke Welsh Corgi and Golden Retrievers, has very few binding sites for the WT1 activator compared to the other major repeat alleles (11, 16*1 and 17). The CAGTGGc motif present in the 16*2 allele provides a binding site for the WT-1 homolog transcriptional silencer KLF12 (Ap-2rep). It has been hypothesized that KLF-12 silences WT-1-dependent gene expression [54]. The decreased binding of WT1 and novel binding of KLF12 to the repeat could decrease *GSTP1* expression. Although there is as yet no experimental evidence of the binding of WT1 and/or KLF12 to the canine *GSTP1* promoter, it would be interesting to establish whether these transcription factors do indeed bind to this repeat sequence and whether the polymorphisms observed affect the number of binding sites and, ultimately, gene expression.

The tandem repeat is located in the 5′UTR of the canine *GSTP1* gene and therefore forms part of the mature mRNA. The microsatellite is also located in proximity to the core promoter (20–30 base pairs downstream of the predicted transcription start site and the TATA box respectively), so variations in the tandem repeats could interfere with the binding of RNA polymerase by changing the spacing between functional elements in promoters [55]. The microsatellite may also affect stability of the secondary structure of the *GSTP1* transcript. In the dog, two types of microsatellites consisting of 16 triplet repeats were observed. The 16*1 allele was predicted to form a more stable RNA hairpin-loop secondary structure than the 16*2 allele. The 16*2 allele alters the structure and position of the hairpin loop, potentially decreasing the stability of the entire *GSTP1* transcript. These hairpin RNA structures may play a role in disease pathogenesis by affecting the processing of the primary transcript, resulting in deficit of the corresponding protein, or interacting with RNA-binding proteins, altering their normal activity [56].

## Conclusions

In summary, this study has demonstrated the existence of multiple polymorphisms in the proximal promoter of the canid *GSTP1* gene. Distinct haplotype differences exist between various dog breeds and between dogs, wolves, coyotes and foxes. The high degree of polymorphism in a 3′UTR microsatellite may be due to replication slippage and unequal allelic recombination events. Some of these variants may affect the binding of transcription factors and/or decrease mRNA stability, altering the detoxification potential of glutathione conjugation catalyzed by GSTP1. In order to investigate any effects that these promoter polymorphisms may have on the glutathione conjugation of xenobiotics, reporter gene assays are underway to determine the effect of the various microsatellite alleles on canine *GSTP1* expression, together with the determination of *GSTP1* mRNA levels and GSTP1 activity in the blood of genotyped dogs.

## Additional files

Additional file 1: Details of individual dogs, wolves, coyotes, and foxes used in the study. (XLSX 20 kb)

Additional file 2: **a** RNA secondary structure of the GCC12*1, 12*2, 16*1, and 16*2 *GSTP1* promoter 5′UTR repeat alleles. **b** Comparison of RNA secondary structure of canine *GSTP1* transcripts with different 5′UTR repeat alleles. (ZIP 191 kb)

Additional file 3: Alignment of dog versus fox *GSTP1* promoter sequences. (DOCX 26 kb)

Additional file 4: Alignment of the dog *GSTP1* 5′UTR with select mammalian *GSTP1* transcripts. (DOCX 158 kb)

Additional file 5: Canid *GSTP1* promoter haplotype and frequencies. (XLSX 16 kb)

Additional file 6: LASAGNA Search results. (XLSX 43 kb)
