# Supplementary figures and images for: Single nucleotide polymorphisms and microsatellites in the canine glutathione S-transferase pi 1 (GSTP1) gene promoter
Source: Canine Genet Epidemiol. 2017 Oct 11;4:9. doi: 10.1186/s40575-017-0050-8 (PMC5635497; doi:10.1186/s40575-017-0050-8)

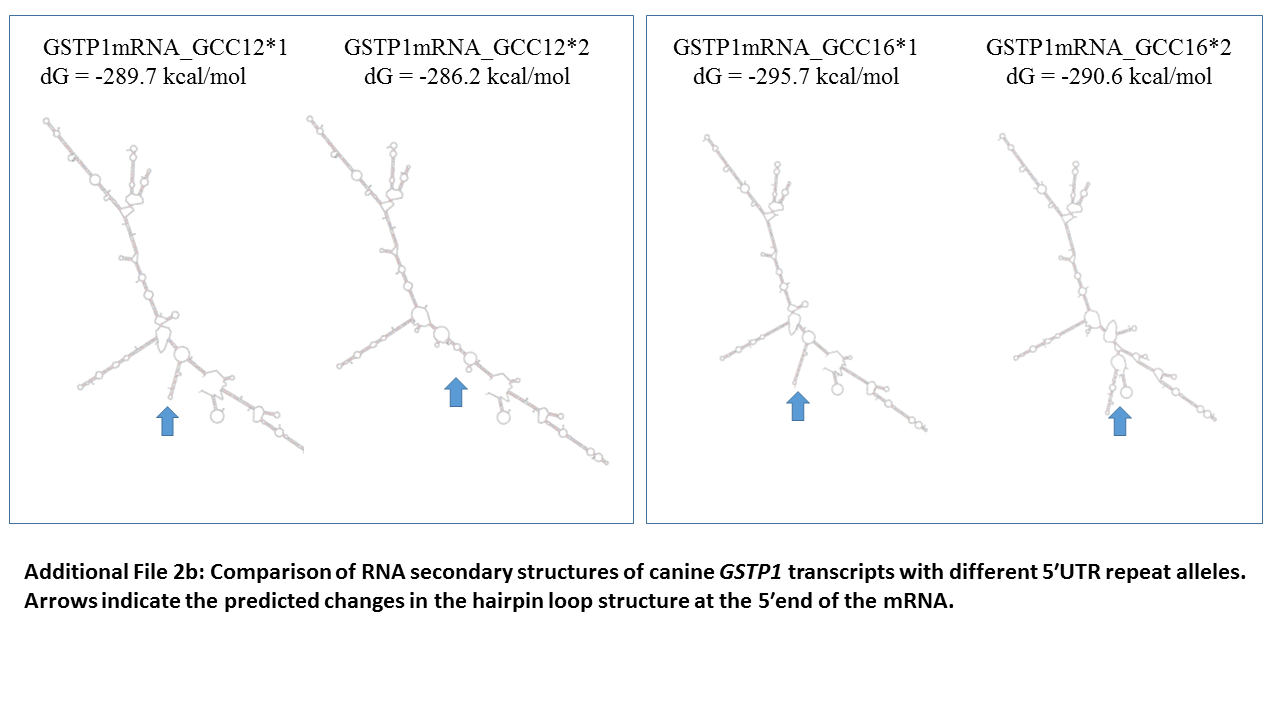

Supplement: Supplementary file 2 — a RNA secondary structure of the GCC12*1, 12*2, 16*1, and 16*2 GSTP1 promoter 5′UTR repeat alleles. b Comparison of RNA secondary structure of canine GSTP1 transcripts with different 5′UTR repeat alleles. (ZIP 191 kb) [file 40575_2017_50_MOESM2_ESM.zip › Additional file 2b_RNA secondary structure.PNG]

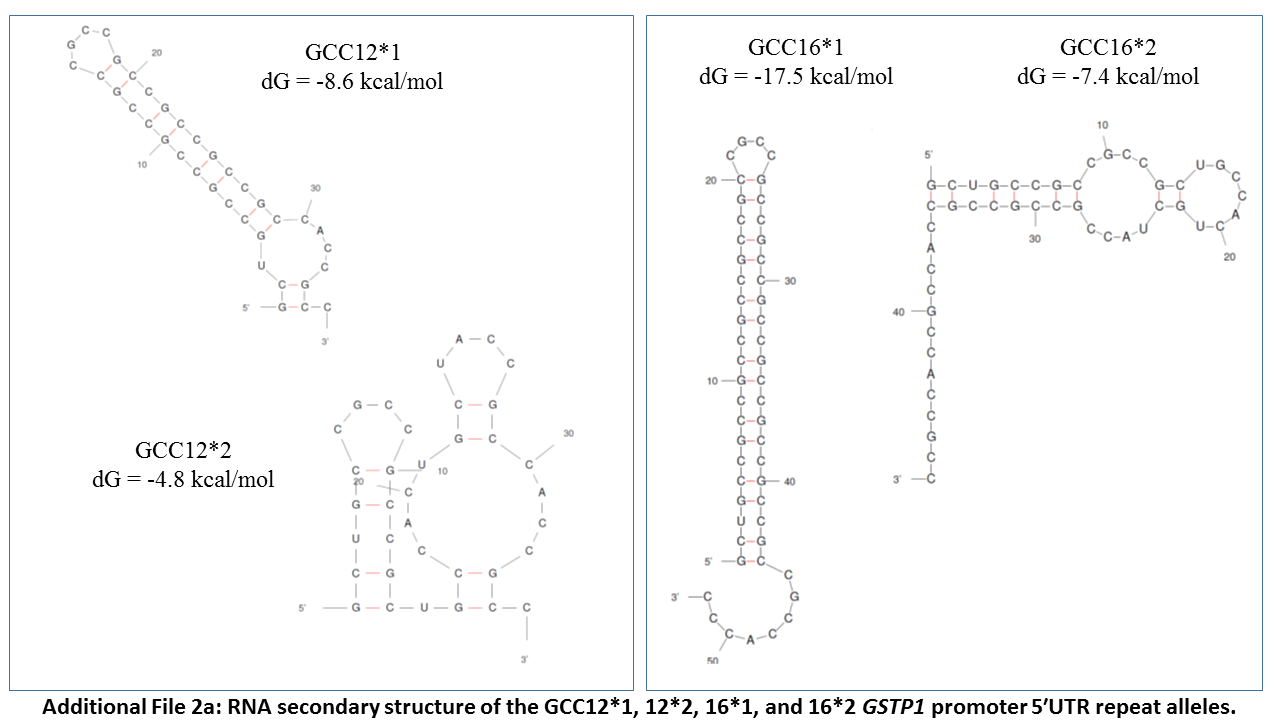

Supplement: Supplementary file 2 — a RNA secondary structure of the GCC12*1, 12*2, 16*1, and 16*2 GSTP1 promoter 5′UTR repeat alleles. b Comparison of RNA secondary structure of canine GSTP1 transcripts with different 5′UTR repeat alleles. (ZIP 191 kb) [file 40575_2017_50_MOESM2_ESM.zip › Additional file 2a_RNA secondary structure.PNG]
