# Supplementary material for: Single nucleotide polymorphisms and microsatellites in the canine glutathione S-transferase pi 1 (GSTP1) gene promoter
Source: Canine Genet Epidemiol. 2017 Oct 11;4:9. doi: 10.1186/s40575-017-0050-8 (PMC5635497; doi:10.1186/s40575-017-0050-8)
Supplement: Supplementary file 3 — Alignment of dog versus fox GSTP1 promoter sequences. (DOCX 26 kb) [file 40575_2017_50_MOESM3_ESM.docx]

**Additional file 3a. Alignment of dog versus red fox *GSTP1* promoter sequences**

BLASTP 2.1.2

Reference: Wise2 Package, Ewan Birney

BLAST like format to play well with existing parsers. Other options are available

See help on the program that generated this for other options

Query= REDFOX_GSTP1PR

Search.................................done

Score E

Sequences producing significant alignments: (bits) Value

DOG_GSTP1pr 891.43 0

>DOG_GSTP1pr

Length = 673 Reversed 0

Score = 891.4 bits (308947), Expect = 0

Query: 3 GGTGTTTCTGTCTAACCCCCTGGCGTCGCCCAGGGTTTCACCACAGCTT 51

D GGTGTTTCTGTCTAACCCCCTGG GTCGCCCAGGGTTTCACCACAGCTT

Sbjct: 3 GGTGTTTCTGTCTAACCCCCTGGTGTCGCCCAGGGTTTCACCACAGCTT 51

Query: 52 GCCTGTCTCCAATAATGTTGCTTTGCTATTCCTGAATAAACCCAATTTG 100

D GC TGTCTCCAATAATG TGCTTTGCTATTCCTGAATAAACCCA TTTG

Sbjct: 52 GCTTGTCTCCAATAATGCTGCTTTGCTATTCCTGAATAAACCCATTTTG 100

Query: 101 TTGGTAAAATAATTGCCTGTTCTGTTGTGAAGGTGGACAAAGGGTTTGT 149

D TGGTAAAATAATTGCCTGTTCTGTTGTGAAGGTGGACAAAGGGTTTGT

Sbjct: 101 CTGGTAAAATAATTGCCTGTTCTGTTGTGAAGGTGGACAAAGGGTTTGT 149

Query: 150 GCCTCTGAGGTCTGACAACCAAGCAGGGCTAGAGGCTGCAATCGGCAAA 198

D GCCTCTGAGGTCTGACAACCAAGCAGGGCTAGAGGCTGCAATCGGCAAA

Sbjct: 150 GCCTCTGAGGTCTGACAACCAAGCAGGGCTAGAGGCTGCAATCGGCAAA 198

Query: 199 CCCCAAGGCCCCTGGGAGCTTCGGCAGCGGGTGCCCTCGCCCTCGCCCC 247

D CCCCAAGGCCCC G AGCTTCGGCAGC GGTGCCCTCGCCCTCGCCC

Sbjct: 199 CCCCAAGGCCCCCGAAAGCTTCGGCAGCTGGTGCCCTCGCCCTCGCCCT 247

Query: 248 CGCCCCCGCCCTCGAGCCCCCAGCGCCAGGCCGAGGATCCGCACGTCCT 296

D CGCCC CGCC CGAGCCCCCAGCGCCAGGCCGAGGATCCGCACGTCCT

Sbjct: 248 CGCCCTCGCCAGCGAGCCCCCAGCGCCAGGCCGAGGATCCGCACGTCCT 296

Query: 297 GCGGGAGGGCGGCGGGCGCAGGGGGGCGGGGGGCTCGGGCGGGGCGGGG 345

D GC GAGGGCGGCGGGCGCAGGGGGGCGGGGGGC C CGGG CGGGG

Sbjct: 297 GCCCGAGGGCGGCGGGCGCAGGGGGGCGGGGGGC-CT--CGGG-CGGGG 341

Query: 346 GGCGCCGGCAGGGTTCGGGTCCCGCAGGGGAGTGGGGAGCCGGACTTCC 394

D GGCGCC GCAGGGTTCGGGTCCCGCAGGGGAGTGGGGAGCCGGACTTCC

Sbjct: 342 GGCGCCAGCAGGGTTCGGGTCCCGCAGGGGAGTGGGGAGCCGGACTTCC 390

Query: 395 GGAGCGCGGTGAGTCAGCACTGGGGCGGGACGGGGCGGAACGGGACGGG 443

D GGAGCGCGGTGA TCAGCACTGGGGCGGGACGGGGCGGAACGGGACGGG

Sbjct: 391 GGAGCGCGGTGACTCAGCACTGGGGCGGGACGGGGCGGAACGGGACGGG 439

Query: 444 GTGGGAGGGACGACCCTTAATAAGGCTGGAGGCCTGGCCGGGGCCTGAG 492

D GTGGGAGGGACGACCCTTAATAAGGCTGGAGGCCTGGCCGGGGCCTGAG

Sbjct: 440 GTGGGAGGGACGACCCTTAATAAGGCTGGAGGCCTGGCCGGGGCCTGAG 488

Query: 493 CTCTGCCGCCGCCGCC 508

D CTCTGC GCCGCCGCC

Sbjct: 489 CTCTGCTGCCGCCGCC 504

>-----------<

Query: 510 CCGCCGCCGCCACCGCCACCGCCACCCGCGCTGCAACCAGTGAGTCCCT 558

D CCGCCGCCGCC CCGCCACCGCCACCCGCGCTGCAACCAGTGAGTCCCT

Sbjct: 521 CCGCCGCCGCCGCCGCCACCGCCACCCGCGCTGCAACCAGTGAGTCCCT 569

Query: 559 GCCGCCTGGGCCTGCTTGGGGAGGGGCTGGTGCTCCCGGGG 599

D GCCGCCTGGGCCTGCTTGGGGAGGGGCTG TGCTCCCGGGG

Sbjct: 570 GCCGCCTGGGCCTGCTTGGGGAGGGGCTGATGCTCCCGGGG 610

Differences from the reference canine sequence are highlighted in yellow. Exon 1 in the dog is underlined.

**Additional file 3b. Alignment of dog versus gray fox *GSTP1* promoter sequences**

BLASTP 2.1.2

Reference: Wise2 Package, Ewan Birney

BLAST like format to play well with existing parsers. Other options are available

See help on the program that generated this for other options

Query= GRAYFOX_GSTP1PR

Search.................................done

Score E

Sequences producing significant alignments: (bits) Value

DOG_GSTP1PR 923.17 0

>DOG_GSTP1PR

Length = 647 Reversed 0

Score = 923.2 bits (319947), Expect = 0

Query: 1 CTCCCTCCTTTTTCTCCGGTGGTGTTTCTGTCTAACCCCCTGGCGTCGC 49

D CTCCCTCCTTTTTCTCCGG GGTGTTTCTGTCTAACCCCCTGG GTCGC

Sbjct: 1 CTCCCTCCTTTTTCTCCGGCGGTGTTTCTGTCTAACCCCCTGGTGTCGC 49

Query: 50 CCAGGGTTTCACCACAGCTTGCTTGTCTCCAATAATGCTGCTTTGCTCT 98

D CCAGGGTTTCACCACAGCTTGCTTGTCTCCAATAATGCTGCTTTGCT T

Sbjct: 50 CCAGGGTTTCACCACAGCTTGCTTGTCTCCAATAATGCTGCTTTGCTAT 98

Query: 99 TCCTGAATAAACCCATTTTGCTGGTAAAATAATTGCCTGTTCTGTTGTG 147

D TCCTGAATAAACCCATTTTGCTGGTAAAATAATTGCCTGTTCTGTTGTG

Sbjct: 99 TCCTGAATAAACCCATTTTGCTGGTAAAATAATTGCCTGTTCTGTTGTG 147

Query: 148 AAGGTGGACAAAGGGTTTGTGCCTCTGAGGTCTGACAACCAAGCAGGGC 196

D AAGGTGGACAAAGGGTTTGTGCCTCTGAGGTCTGACAACCAAGCAGGGC

Sbjct: 148 AAGGTGGACAAAGGGTTTGTGCCTCTGAGGTCTGACAACCAAGCAGGGC 196

Query: 197 TAGAGGCTGCAATCGGCAAACCCCAAGGCCCTGGAA-GCTTCGGCAGCT 244

D TAGAGGCTGCAATCGGCAAACCCCAAGGCCC GAA GCTTCGGCAGCT

Sbjct: 197 TAGAGGCTGCAATCGGCAAACCCCAAGGCCCCCGAAAGCTTCGGCAGCT 245

Query: 245 GGTGCCCTCGCCCTCGCC 262

D GGTGCCCTCGCCCTCGCC

Sbjct: 246 GGTGCCCTCGCCCTCGCC 263

>-----------<

Query: 264 GCGAGCCCCCAGCGCCAGGCCGAGGATGCGCACGTCCTGCCGGAGGGCG 312

D GCGAGCCCCCAGCGCCAGGCCGAGGAT CGCACGTCCTGCC GAGGGCG

Sbjct: 277 GCGAGCCCCCAGCGCCAGGCCGAGGATCCGCACGTCCTGCCCGAGGGCG 325

Query: 313 GCGGGCGCAGGGGGGCGGGGGGC 335

D GCGGGCGCAGGGGGGCGGGGGGC

Sbjct: 326 GCGGGCGCAGGGGGGCGGGGGGC 348

>-----------<

Query: 337 CCAGCGGGGTTCGGGTCCTGCAGGGGAGTGGGGAGCCGGACTTCCGGAG 385

D CCAGC GGGTTCGGGTCC GCAGGGGAGTGGGGAGCCGGACTTCCGGAG

Sbjct: 364 CCAGCAGGGTTCGGGTCCCGCAGGGGAGTGGGGAGCCGGACTTCCGGAG 412

Query: 386 CGCGGTGAGTCAGCACTGGGGCGGGACGGGGCGGAACGGGACGGGGTGG 434

D CGCGGTGA TCAGCACTGGGGCGGGACGGGGCGGAACGGGACGGGGTGG

Sbjct: 413 CGCGGTGACTCAGCACTGGGGCGGGACGGGGCGGAACGGGACGGGGTGG 461

Query: 435 GAGGGACGACCCTTAATAAGGCTGGAGGCCTGGCCGGGGCCTGAGCTCT 483

D GAGGGACGACCCTTAATAAGGCTGGAGGCCTGGCCGGGGCCTGAGCTCT

Sbjct: 462 GAGGGACGACCCTTAATAAGGCTGGAGGCCTGGCCGGGGCCTGAGCTCT 510

Query: 484 GCTGCCGCCGCCGCCGCCGC 503

D GCTGCCGCCGCCGCCGCCGC

Sbjct: 511 GCTGCCGCCGCCGCCGCCGC 530

>-----------<

Query: 505 ACCGCCACCCGCGCTGCAACCAGTGAGTCCCTGGCGCCTGGGCCTGCTT 553

D ACCGCCACCCGCGCTGCAACCAGTGAGTCCCTG CGCCTGGGCCTGCTT

Sbjct: 556 ACCGCCACCCGCGCTGCAACCAGTGAGTCCCTGCCGCCTGGGCCTGCTT 604

Query: 554 GGGGAGGGGCTGATGCTCCCGGGGGCCTCGGAATGCTCCCGG 595

D GGGGAGGGGCTGATGCTCCCGGGGGCCTCGGAATGCTCCCGG

Sbjct: 605 GGGGAGGGGCTGATGCTCCCGGGGGCCTCGGAATGCTCCCGG 646

Differences from the reference canine sequence are highlighted in yellow. Exon 1 in the dog is underlined.

**Additional file 3c. Multiple alignment of dog, red fox, and grey fox *GSTP1* promoter sequences**

# Percent Identity Matrix - created by Clustal2.1

#

1: GREYFOX_GSTP1PR 100.00 98.15 96.14

2: DOG_GSTP1pr 98.15 100.00 95.89

3: REDFOX_GSTP1pr 96.14 95.89 100.00

CLUSTAL O(1.2.4) multiple sequence alignment (DOG EXON 1 IN BOLD AND UNDERLINED)

GREYFOX_GSTP1PR CTCCCTCCTTTTTCTCCGGTGGTGTTTCTGTCTAACCCCCTGGCGTCGCCCAGGGTTTCA 60

DOG_GSTP1PR CTCCCTCCTTTTTCTCCGGCGGTGTTTCTGTCTAACCCCCTGGTGTCGCCCAGGGTTTCA 60

FOX_GSTP1pr CTCCCTCCTTTTTCTCCGGTGGTGTTTCTGTCTAACCCCCTGGCGTCGCCCAGGGTTTCA 60

******************* *********************** ****************

GREYFOX_GSTP1PR CCACAGCTTGCTTGTCTCCAATAATGCTGCTTTGCTCTTCCTGAATAAACCCATTTTGCT 120

DOG_GSTP1PR CCACAGCTTGCTTGTCTCCAATAATGCTGCTTTGCTATTCCTGAATAAACCCATTTTGCT 120

FOX_GSTP1pr CCACAGCTTGCCTGTCTCCAATAATGTTGCTTTGCTATTCCTGAATAAACCCAATTTGTT 120

*********** ************** ********* **************** **** *

GREYFOX_GSTP1PR GGTAAAATAATTGCCTGTTCTGTTGTGAAGGTGGACAAAGGGTTTGTGCCTCTGAGGTCT 180

DOG_GSTP1PR GGTAAAATAATTGCCTGTTCTGTTGTGAAGGTGGACAAAGGGTTTGTGCCTCTGAGGTCT 180

FOX_GSTP1pr GGTAAAATAATTGCCTGTTCTGTTGTGAAGGTGGACAAAGGGTTTGTGCCTCTGAGGTCT 180

************************************************************

GREYFOX_GSTP1PR GACAACCAAGCAGGGCTAGAGGCTGCAATCGGCAAACCCCAAGGCCCTGGA-AGCTTCGG 239

DOG_GSTP1PR GACAACCAAGCAGGGCTAGAGGCTGCAATCGGCAAACCCCAAGGCCCCCGAAAGCTTCGG 240

FOX_GSTP1pr GACAACCAAGCAGGGCTAGAGGCTGCAATCGGCAAACCCCAAGGCCCCTGGGAGCTTCGG 240

*********************************************** * ********

GREYFOX_GSTP1PR CAGCTGGTGCCCTCGCCCT------------CGCCAGCGAGCCCCCAGCGCCAGGCCGAG 287

DOG_GSTP1PR CAGCTGGTGCCCTCGCCCTCGCCCTCGCCCTCGCCAGCGAGCCCCCAGCGCCAGGCCGAG 300

FOX_GSTP1pr CAGCGGGTGCCCTCGCCCTCGCCCCCGCCCCCGCCCTCGAGCCCCCAGCGCCAGGCCGAG 300

**** ************** **** ***********************

GREYFOX_GSTP1PR GATGCGCACGTCCTGCCGGAGGGCGGCGGGCGCAGGGGGGCGGGGGGC------------ 329

DOG_GSTP1PR GATCCGCACGTCCTGCCCGAGGGCGGCGGGCGCAGGGGGGCGGGGGGCC----TCGGGCG 356

FOX_GSTP1pr GATCCGCACGTCCTGCGGGAGGGCGGCGGGCGCAGGGGGGCGGGGGGCTCGGGCGGGGCG 360

*** ************ ******************************

GREYFOX_GSTP1PR ------GCCAGCGGGGTTCGGGTCCTGCAGGGGAGTGGGGAGCCGGACTTCCGGAGCGCG 389

DOG_GSTP1PR GGGGGCGCCAGCAGGGTTCGGGTCCCGCAGGGGAGTGGGGAGCCGGACTTCCGGAGCGCG 416

FOX_GSTP1pr GGGGGCGCCGGCAGGGTTCGGGTCCCGCAGGGGAGTGGGGAGCCGGACTTCCGGAGCGCG 402

*** ** ************ **********************************

GREYFOX_GSTP1PR GTGAGTCAGCACTGGGGCGGGACGGGGCGGAACGGGACGGGGTGGGAGGGACGACCCTTA 449

DOG_GSTP1PR GTGACTCAGCACTGGGGCGGGACGGGGCGGAACGGGACGGGGTGGGAGGGACGACCCTTA 476

FOX_GSTP1pr GTGAGTCAGCACTGGGGCGGGACGGGGCGGAACGGGACGGGGTGGGAGGGACGACCCTTA 480

**** *******************************************************

GREYFOX_GSTP1PR ATAAGGCTGGAGGCCTGGCCGGGGCCTGAGCTCTGCTGCCGCCGCCGCCGCC-------- 498

DOG_GSTP1PR ATAAGGCTGGAGGCCT**GGCCGGGGCCTGAGCTCTGCTGCCGCCGCCGCCGCCGCTGCCGC** 536

FOX_GSTP1pr ATAAGGCTGGAGGCCTGGCCGGGGCCTGAGCTCTGCCGCCGCCGCCACCGCC-------- 532

************************************ ********* *****

GREYFOX_GSTP1PR ----------------GCCACCGCCACCCGCGCTGCAACCAGTGAGTCCCTGGCGCCTGG 545

DOG_GSTP1PR **CGCCGCCGCCGCCGCCGCCACCGCCACCCGCGCTGCAACCA**GTGAGTCCCTGCCGCCTGG 596

FOX_GSTP1pr -------GCCGCCACCGCCACCGCCACCCGCGCTGCAACCAGTGAGTCCCTGCCGCCTGG 585

************************************ *******

GREYFOX_GSTP1PR GCCTGCTTGGGGAGGGGCTGATGCTCCCGGGGGCCTCGGAATGCTCCCGGC 596

DOG_GSTP1PR GCCTGCTTGGGGAGGGGCTGATGCTCCCGGGGGCCTCGGAATGCTCCCGGC 647

FOX_GSTP1pr GCCTGCTTGGGGAGGGGCTGGTGCTCCCGGGGTCCTCGGAATGCTCCCGGC 636

******************** *********** ******************
