# Supplementary material for: Single nucleotide polymorphisms and microsatellites in the canine glutathione S-transferase pi 1 (GSTP1) gene promoter
Source: Canine Genet Epidemiol. 2017 Oct 11;4:9. doi: 10.1186/s40575-017-0050-8 (PMC5635497; doi:10.1186/s40575-017-0050-8)

**Additional file 4. Alignment of the dog *GSTP1* 5'UTR with select mammalian *GSTP1* transcripts**

Sequences selected all included at least a 50-nt length in the 5'UTR.

Key:-

| Nucleotide accession number | Species | Common name | Order |
| --- | --- | --- | --- |
| NM_012577.2 | *Rattus norvegicus* | Brown rat | Rodentia |
| NM_013541.1 | *Mus musculus* | House mouse | Rodentia |
| NM_000852.3 | *Homo sapiens* | Human | Primates |
| XM_010366328.1 | *Rhinopithecus roxellana* | Golden snub-nosed monkey | Primates |
| XM_017857325.1 | *Rhinopithecus bieti* | Black snub-nosed monkey | Primates |
| XM_011287131.1 | *Felis catus* | Domestic cat | Carnivora |
| XM_019812765.1 | *Felis catus (variant X2)* | Domestic cat | Carnivora |
| NM_001252167.1 | *Canis lupus familiaris* | Domestic dog | Carnivora |
| XM_006743604.1 | *Leptonychotes weddellii* | Weddell Seal | Carnivora |
| XM_004759714.2 | *Mustela putorius furo* | Ferret | Carnivora |
| XM_014844695.1 | *Equus asinus* | Wild Ass | Perissodactyla |
| XM_001498106.5 | *Equus caballus* | Horse | Perissodactyla |
| XM_015238625.1 | *Vicugna pacos* | Alpaca | Artiodactyla |
| XM_010956739.1 | *Camelus bactrianus* | Bactrian Camel | Artiodactyla |
| XM_010998117.1 | *Camelus dromedarius* | Dromedary | Artiodactyla |
| XM_020880767.1 | *Odocoileus virginianus texanus* | White-tailed deer | Artiodactyla |
| XM_019955248.1 | *Bos indicus* | Zebu | Artiodactyla |
| NM_177516.1 | *Bos taurus* | Cattle | Artiodactyls |
| XM_018043050.1 | *Capra hircus* | Goat | Artiodactyla |
| XM_012117476.1 | Ovis aries musimon | Mouflon | Artiodactyla |

Alignment of entire transcript computed by ClustalOmega. Only actual or predicted 5’UTR portion of the sequence shown. Canine microsatellite highlighted in yellow; potential microsatellites or GCC repeats in other species are shown color coded according to species order. Bold sequence: dog; Underlined sequence: start codon. Regions of zero homology are indicated by dashed lines.

CLUSTAL O(1.2.4) multiple sequence alignment

NM_012577.2 -------ATTCGTCTGCGTCTGAGATACTTCATCGTCCACGCAGCTTTGAGTCCACACCT 53

NM_013541.1 ----------------------------CTCTGAGTACCCCTCTGTCTACGCAGCACTGA 32

NM_000852.3 GGCCGCGA------------------GGCCTTCGCTGGAGTTTCGCCGCCG--------- 235

XM_010366328.1 GGCCGCAA------------------GGCCTGCGCTGGAGTTTCGTCGTCCCCGCCGCCG 203

XM_017857325.1 GGCCGCAA------------------GGCCTGCGCTGGAGTTTCGTCGTCCCCGCCGCCG 283

XM_011287131.1 --------------------------------GCCGCCGCCACCGCCGCCTGAGCCGCCG 28

XM_019812765.1 --------------------------------GCCGCCGCCACCGCCGCCTGAGCCGCCG 28

**NM_001252167.1 GCCTGAGCTCTGCTGCCGCCGCCGCCGCCGCTGCCGCCGCCGCCGCCGCCACCGCCACCG 67**

XM_006743604.1 CGAGGGCGGAACGACGGGCCTGAGCTGTGCCGCCGACCTCCGCCGCCGCCGCCGCCGTCT 83

XM_004759714.2 ---------------------------------------CCACCGCCACTGCCACCCCCG 21

XM_014844695.1 --------GCCTCCAGGCAGCAGGCTTGAAGTTCCGCTGCCGCCGCCGCCGCCTGAGCCG 355

XM_001498106.5 --------GCTTCCAGGCAGCAGGCTTGAAGTTCCGCTGCCGCCGCCGCCGCCTGAGCCG 87

XM_015238625.1 ----------------------------GAGCTCTGCTGCCGCTGCTGCCGCCGTGACCA 32

XM_010956739.1 ------------------CTGCCGCCGCTGCCGCCGCTGCCGCCGCTGCCGCCGTGACCA 42

XM_010998117.1 GCCGCCAGTCGGAGCGCTCGAGCTCTGCTGCCGCCGCTGCCGCCGCTGCCGCCGTGACCA 180

XM_020880767.1 C----------TGGAGTTTTGCCGCCGCCGCCGCCGCCACCGCCACCGCCGCCTTTGCAG 53

XM_019955248.1 C----------CGCCGCCSAGCGCGCTGGAGCTTTG---CTGCCGCCGCCACCTTTACCG 54

NM_177516.1 C----------CGCCGCCGAGCGCGCTGGAACTTTG---CTGCCGCCGCCACCTTTACCG 56

XM_018043050.1 C----------AGCCGCCGAGCGCGCTGGAGTTTTGCCGCC---GCCGCCACATTTACCG 344

XM_012117476.1 C----------AGCCGCCGAGCGCGCTGGAGTTTTGCCGCCGCCGCCGCCACGTTTACCG 105

NM_012577.2 CTGTCTACGCAGCAGCTATGCCACCGTACACCATTGTGTACTTCCCAGTTCGAGGGCGCT 113

NM_013541.1 ATCCGCACCCAGCAGGCATGCCACCATACACCATTGTCTACTTCCCAGTTCGAGGGCGGT 92

NM_000852.3 ---CAGTCTTCGCCACCATGCCGCCCTACACCGTGGTCTATTTCCCAGTTCGAGGCCGCT 292

XM_010366328.1 CCGCCGCGTGTGCCATCATGCCGCCCTACACCGTGGTCTACTTCCCAGTTCGAGGCCGCT 263

XM_017857325.1 CCGCCGCGTGTGCCATCATGCCGCCCTACACCGTGGTCTACTTCCCAGTTCGAGGCCGCT 343

XM_011287131.1 CTGTCCGCGCTGCAACCATGCCGCCCTACACCATTGTCTACTTCCCGGTCCGAGGGCGCT 88

XM_019812765.1 CTGTCCGCGCTGCAACCATGCCGCCCTACACCATTGTCTACTTCCCGGTCCGAGGGCGCT 88

**NM_001252167.1 CCACCCGCGCTGCAACCATGCCACCCTACACCATCACCTACTTCCCTGTTCGAGGGCGCT 127**

XM_006743604.1 CCATCCGCGTTGCAACCATGGCGCCCTACACCATTGTCTACTTTCCTGTGCGAGGGCGCT 143

XM_004759714.2 CCACCCGCGCTGCAACCATGCCGCCCTACACCATTGTCTACTTTCCTGTCCGAGGCCGCT 81

XM_014844695.1 CTGTCTACACTGCAACGATGCCGCCCTACACCATCGTCTACTTCTCCGTTCGAGGGCGCT 415

XM_001498106.5 CTGTCTACACTGCAACGATGCCGCCCTACACCATCGTCTACTTCTCCGTTCGAGGGCGCT 147

XM_015238625.1 CCATCCCTGCCGCCACCATGCCGCCCTACACCATTGTCTACTTCCCTGTTCGAGGGCGCT 92

XM_010956739.1 CCGTCCCTGCCGCCACCATGCCGCCCTACACCATTGTCTACTTCCCTGTTCGAGGGCGCT 102

XM_010998117.1 CCGTCCCTGCCGCCACCATGCCGCCCTACACCATTGTCTACTTCCCTGTTCGAGGGCGCT 240

XM_020880767.1 ACTTCCCCGTCGCCAGGATGCCGCCCTACACCATTGTCTACTTCCCGGTTCAAGGGCGCT 113

XM_019955248.1 ACTTCCCCGACTCCAGGATGCCTCCCTACACCATCGTCTACTTCCCGGTTCAAGGGCGCT 114

NM_177516.1 ACTTCCCCGACTCCAGGATGCCTCCCTACACCATCGTCTACTTCCCGGTTCAAGGGCGCT 116

XM_018043050.1 ACTTCCCCGTCGCCAGGATGCCGCCCTACACCATCGTCTACTTCCCGGTTCAAGGGCGCT 404

XM_012117476.1 ACTTCCCCGTCGCCAGGATGCCGCCCTACACCATCGTCTACTTCCCGGTTCAAGGGCGCT 165

* *** * ** ****** * ** ** * ** * *** ** *


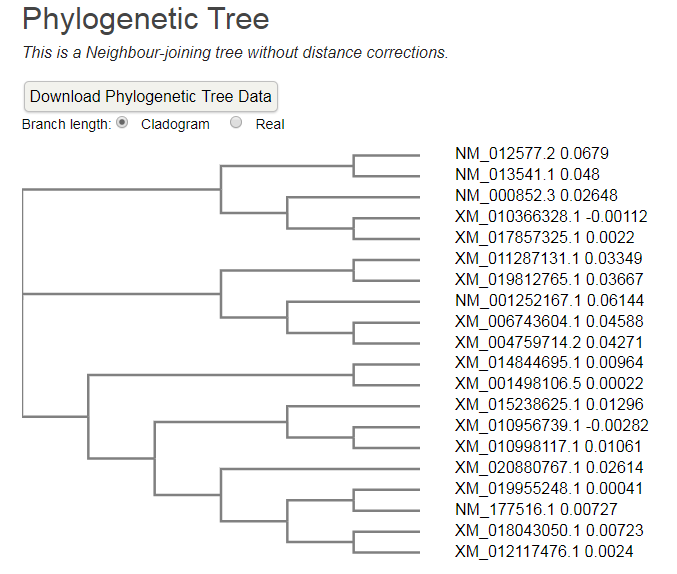


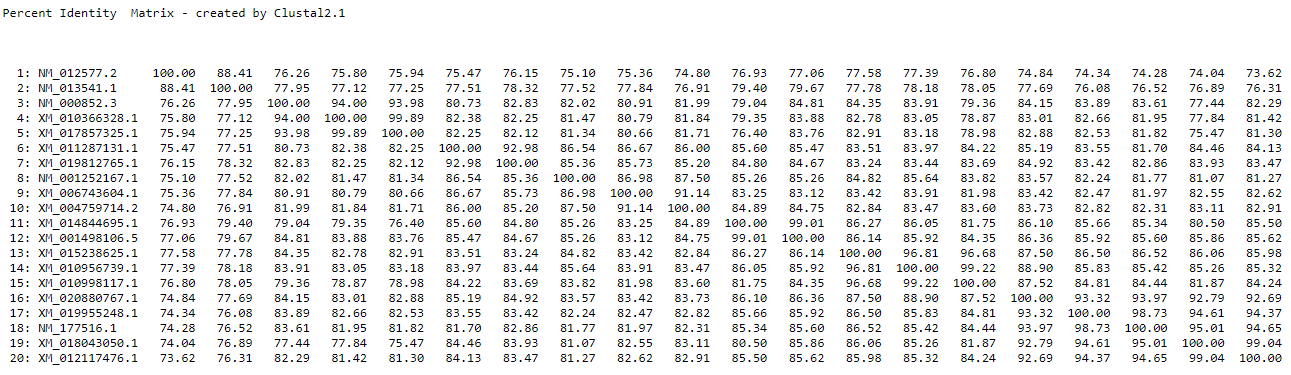

Supplement: Supplementary file 4 — Alignment of the dog GSTP1 5′UTR with select mammalian GSTP1 transcripts. (DOCX 158 kb) [file 40575_2017_50_MOESM4_ESM.docx]
